# Preventing Internalizing Problems in Young Children: A Randomized Controlled Trial of the Feelings and Friends (Year 3) Program with a Motor Skills Component

**DOI:** 10.3389/fpsyg.2017.00291

**Published:** 2017-03-07

**Authors:** Ruhamah G. Tennant, Katie K. Martin, Rosanna Rooney, Sharinaz Hassan, Robert T. Kane

**Affiliations:** Faculty of Health Sciences, School of Psychology and Speech Pathology, Curtin UniversityPerth, WA, Australia

**Keywords:** Aussie Optimism, children, mental health problems, motor skills, intervention studies

## Abstract

Anxiety and depression are common mental health problems experienced by children in Australia. The impact of these internalizing disorders is pervasive, affecting many areas of life. By the time problems have been detected in children they can be severe in nature and harder to treat. Hence, early intervention is of utmost importance. Despite the existence of numerous prevention programs for children, there is limited empirical evidence for a program that has an impact on symptoms of both anxiety and depression. Physical activity and improved motor coordination have been indicated as having positive effects on children's mental health, although the impact of including these in a program targeting internalizing disorders has not been established. This study aimed to evaluate the efficacy of the Feelings and Friends (Year 3) program (FFY3), revised to include activities to build motor-coordination and encourage physical activity. Participants were 24 children from the Perth metropolitan area alongside one of each of their parents. Results indicated significant short-term intervention effects on one of the primary outcome variables; intervention group parents reported significant pre-post improvement in child depressive symptoms, which were maintained at 3-month follow-up (η_*p*_^2^ = 0.10). There were also intervention effects observed for parent-reported separation anxiety (η_*p*_^2^ = 0.10), externalizing symptoms (η_*p*_^2^ = 0.19), and conduct problems (η_*p*_^2^ = 0.16). An additional finding indicated the intervention students reported significant improvement from session one to session two in global distress (η_*p*_^2^ = 0.22). No other significant intervention effects were evident. Findings from this study indicate that FFY3 is a promising intervention to address internalizing and externalizing symptoms in 8–9 year-old children.

## Introduction

Internalizing disorders refer to emotional distress and the emotional symptoms associated with anxiety and depression (Bayer et al., 2011). In Australia, anxiety disorders occur in children at a rate of 6.9% and depressive disorders at a rate of 2.8% (Lawrence et al., 2015). The impact of emotional problems on children includes reduced adaptive functioning, interpersonal and relationship difficulties, academic problems, lowered self-esteem and social competence deficits (Barrett et al., 2005). By the time internalizing problems have been detected in children, problems can be severe in nature and thus harder to treat, potentially leading to long-term impacts extending beyond the symptoms of a disorder (Bayer et al., 2011). Consequently, early intervention is of utmost importance and empirically supported programs are required.

Prevention programs typically take two forms, those that are universal, where the intervention is delivered to a population regardless of risk, and those that are targeted, where the intervention is only delivered to those at high risk of developing a psychological disorder (Merry et al., 2012). The advantages of employing a universal approach include avoiding stigma that may result from participants being labeled when included in a targeted intervention. Universal delivery also ensure that children who are “at risk” are not overlooked, and children who have skills in the target area can be models and peer supporters (Lowry-Webster et al., 2001; Kösters et al., 2012). When children learn in a group environment they are encouraged to articulate their thoughts, express and manage their emotions, and display respect and empathy to the feelings of others (Johnson and Johnson, 2009; Denham and Brown, 2010). The development of these skills is likely to complement the prevention of the internalizing problems.

Programs aiming to prevent internalizing disorders in young children have typically focused on the development of social-emotional competence, which encompasses awareness and regulation of emotions, perspective taking, and problem-solving skills (Domitrovich et al., 2007). These skills are considered developmentally appropriate for young children (Durlak et al., 1991). Empirical research on the efficacy of programs developed to enhance social-emotional competence (e.g., Kusche and Greenberg, 1994; Bernard, 2004) has shown reductions in child-reported aggression and depressive symptoms (*d* = 0.49) and reductions in teacher-reported internalizing (*d* = 0.22) and externalizing behaviors (*d* = 0.18). There have also been significant positive effects on social-emotional competence (η^2^ = 0.22), social skills (η^2^ = 0.32), and wellbeing (η^2^ = 0.16) and reported increases in child and teacher reported resilience and affective vocabulary (*d* = 0.54) (Kam et al., 2004; Ashdown and Bernard, 2012; Crean and Johnson, 2013). Some of these programs are limited by their delivery over an extended period (e.g., Kusche and Greenberg, 1994), and some research has relied on a clinical population to demonstrate efficacy (e.g., Bernard, 2004) or was conducted in one school (e.g., Ashdown and Bernard, 2012) limiting the generalizability of findings.

Researchers have also focused on evaluating the effectiveness of numerous cognitive-behavior therapy based prevention programs (e.g., Dadds et al., 1997; Barrett and Turner, 2001; Lyneham et al., 2003; Hirshfeld-Becker et al., 2008). All of these programs were modeled on the Coping Cat program (Kendall P. et al., 1992) and utilize techniques such as relaxation, cognitive restructuring, and *in vivo* exposure to address internalizing symptoms. The programs vary in group size, length (8–12 weeks), and the inclusion/exclusion criteria for children to participate in the intervention. Results from clinical trials include significant reductions in anxiety disorders (*d* = 0.55; Hirshfeld-Becker et al., 2010); lowered anxiety symptomology at 12-month follow-up (*d* = 0.32; Lock and Barrett, 2003) and 36-month follow-up (*d* = 0.70) (Barrett et al., 2006); and lowered parent-reported anxiety and emotional symptoms (Hudson et al., 2009). Limitations of this research include a heavy reliance on parent report measures (e.g., Hirshfeld-Becker et al., 2010), with child-reported outcomes not generally showing significant change (e.g., Hudson et al., 2009). In general, the programs focus on anxiety with few studies showing findings for a reduction in depressive symptoms (e.g., Dadds et al., 1997). A meta-analysis of depression prevention programs has shown that on average, intervention effects are small in samples of normative children, however in these studies children still presented with significant reductions in depressive symptomology (Stice et al., 2009). This research highlights the clinical significance of reductions in internalizing symptoms with small effect sizes, in a normative population.

The Feelings and Friends program (Rooney et al., 2014) is a 10-week program incorporating social-emotional, cognitive, and behavioral strategies. This program fills a gap in the existing prevention research by targeting anxiety and depression and teaching social and emotional competence (Rooney et al., 2006). By including behavioral strategies for anxiety management and pleasant event scheduling for depression, children learn ways to cope (Kendall P. C. et al., 1992). An initial trial of the program (Pophillat, 2007) found a significant reduction in parent-reported anxiety symptoms ($\eta_{p}^{2}$ = 0.02). Later when the program was adapted to suit the language skills of children in years 1 and 2, a trial found a significant pre-post reduction in parent-reported anxiety (*r* = 0.89), psychological difficulties (*r* = 0.72), and overall distress (*r* = 0.88). Combined, these studies show that Feelings and Friends holds promise for reducing both anxiety and depressive symptoms.

Physical activity has been shown to reduce depressive and anxious symptoms, psychological distress, and emotional disturbance (*d* = 0.30; Ahn and Fedewa, 2011); however, further investigation regarding the mechanism of change is warranted. Prevention programs have not commonly included physical activity as a mean of reducing internalizing symptoms. Thus, the impact of preventative programs, including Feelings and Friends, could be improved by the inclusion of activities to encourage physical activity in children. A body of research has suggested that improving children's perceived competency in motor ability can increase their participation in physical activity (e.g., (Hay et al., 2004)). Given this relationship, the current study incorporates activities from the Animal Fun program (Piek et al., 2012a), which was designed to improve motor coordination and social-emotional development in young children. Activities involve the imitation of animals with the idea that fun and meaningful actions will encourage children to participate. A randomized controlled trial of the program with 511 children demonstrated significant improvement in motor skills at 18-month follow-up (Piek et al., 2012b).

Fresel (2013) conducted a pilot randomized controlled trial of the Feelings and Friends (Year 1 and 2) program revised to include Animal Fun activities. The major finding was a reduction in parent-reported child depression ($\eta_{p}^{2}$ = 0.18). Although, Animal Fun activities were added to the existing program, no outcome measure for physical activity was utilized. As such, further randomized controlled trials of Feelings and Friends are needed to confirm the efficacy of the program and determine the impact of including motor coordination activities.

## The current study

The current study aimed to extend Fresel's (2013) research by examining the efficacy of the revised Feelings and Friends (Year 3) Program (FFY3) at reducing symptoms of internalizing disorders in children aged 8–9 years. It is the first randomized controlled trial of the Year 3 program. FFY3 will continue to promote emotional awareness and regulation, build social competence and self-efficacy, and teach problem-solving and coping skills, however, this research adds motor-coordination activities to encourage physical activity. FFY3 is designed for universal implementation and thus inclusion does not require a clinical diagnosis. This program retained its fidelity despite the addition of activities and involved the use of multi-informant measures to validate the effectiveness of the intervention. Given past evaluations of the Feelings and Friends program have reported a variety of significant outcomes, it was expected that FFY3 would result in reductions in symptoms of anxiety and depression. The inclusion of activities to develop motor coordination was anticipated to improve participants' perception of their physical competence, thus increasing their engagement in physical activity, with flow on benefit to their mental health. Consequently, it was hypothesized:

H1 (a) Children in the intervention group will report significant pre-post reduction in depression (as measured by the Short Mood and Feelings Questionnaire [SMFQ]); (b) anxiety (as measured by the Spence Child Anxiety Scale [SCAS]); these reductions will be maintained at follow-up, whereas the waitlist control will show no reduction during this period.
H2: Children in the intervention group will report significant pre-post improvements in their physical abilities (as measured by the athletic competence subscale of Self-Perception Scale for Children [SPPC]); these improvements will be maintained at follow-up whereas the waitlist control will show no improvement during this period.
H3: (a) Parents of children in the intervention group will report significant pre-post reduction in child depression (as measured by the Short Mood and Feelings Questionnaire for Parents [SMFQ-P]); (b) anxiety (as measured by the parent version of the Spence Child Anxiety Scale- Parent Version [SCAS-P]); and (c) psychological difficulties (as measured by the total difficulties subscale of the Strengths and Difficulties Questionnaire [SDQ]); these reductions will be maintained at follow-up, whereas parents of children in the waitlist control will report no reduction during this period.

A secondary hypothesis relates to children in the intervention group only:

H4: Children will report significant improvements in well-being (as measured by the Child Outcome Rating Scale [CORS]) over the nine intervention sessions.

## Methods

### Research design

The current study involved a 2 (group; intervention and control) × 3 (time; pre, post, and 3-month follow-up outcome data) randomized controlled trial design. This study extended over 5 months. Approval for the study was obtained from the Curtin University Human Resources Ethics Committee.

### Participants

**Figure 1:**
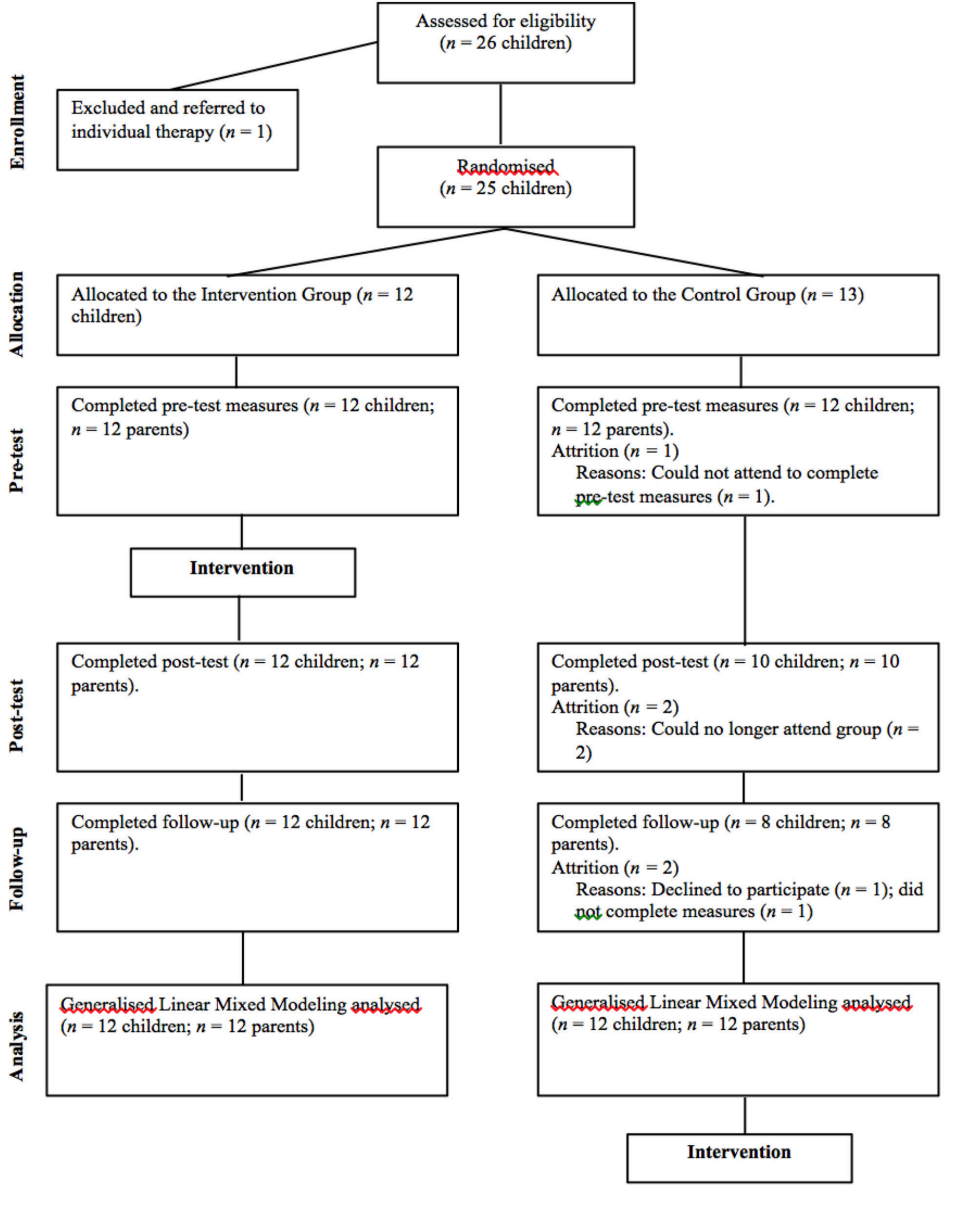
**Diagram illustrating the flow of participants through all stages of the FFY3 randomized controlled trial**.

Participants were 24 children in Year 3 (between 8 and 9 year-old) in the Perth metropolitan area. One parent of each child also participated by completing outcome measures. The flow of participants through each stage of the study is shown in Figure 1. To be eligible for the program, the child had to be a current year 3 student residing in the Perth metropolitan area. As FFY3 is a preventative program, inclusion in the intervention was not based on clinical cut-off scores. An unselected sample was sought whereby any child who wished to be involved in the program was included. The only exception to this inclusion criteria was children whose parents reported severe behavioral difficulties or suicidal ideation. These children were referred on to other services to ensure a harmonious group environment and to limit the potential for distressing disclosures and disruption to group processes and cohesion.

Meta analyses' results where researchers reviewed universal prevention programs for children, suggest there is great heterogeneity in program content, delivery and outcome measures; therefore, the estimation of overall effect sizes is difficult (e.g., Merry et al., 2012). A priori power analysis conducted using G^*^Power (Faul et al., 2009) estimated that in order to have an 80% chance of detecting a moderate interaction (i.e., *f* = 0.25) between group (intervention, control) and time (pre-test, post-test, and follow-up) 36 participants (18 per group) were required. Due to time constraints for the completion of this project the number of participants fell short of this estimate.

### Measures

#### Child-report measures

The Short Mood and Feelings Questionnaire for Children (SMFQ; Angold et al., 1995) is a 13-item scale used to assess cognitive and affective symptoms of depression in children aged 6–17 years. Participants are asked to rate descriptions of how they may have been feeling in the previous 2 weeks (e.g., “I felt miserable or unhappy”) on a 3-point Likert scale (*true, sometimes true*, or *not true*). The scale's internal consistency is reportedly good (α = 0.85) and it shows moderate correlations with other measures of depressive symptoms in children such as the Children's Depression Inventory (CDI; *r* = 0.67; Kovacs, 1992). The internal consistency of the SMFQ in the current study presented as acceptable for research purposes (α = 0.86).

The Spence Children's Anxiety Scale (SCAS; Spence, 1998) is a 45-item questionnaire used to assess symptoms relating to anxiety disorders in children aged 8–12 years. Participants are required to report the presence and frequency of symptoms via a 4-point scale (*never, sometimes, often*, or *always*). Items are scored from 0 to 3 with a clinical cut-off of 42 (Barrett et al., 2005). The SCAS demonstrates excellent internal consistency (α = 0.92). Test-re-test reliability is reportedly satisfactory, with reliability coefficients ranging from 0.60 to 0.63 (Spence; Spence et al., 2003). In addition, the SCAS has good convergent validity and significant correlation with Reynolds and Richmond's (1997) Revised Children's Manifest Anxiety Scale (*r* = 0.75) and divergent validity shown in the weaker correlation with the CDI (*r* = 0.60) (Spence et al., 2003). The internal consistency of the SCAS in the current study is good (α = 0.91).

The Self-Perception Profile for Children (SPPC; Harter, 2012) is a 36-item questionnaire used to assess self-concept in children aged 8–15 years. The measure comprises six subscales (i.e., scholastic competence, social competence, athletic competence, physical appearance, behavioral conduct, and global self-worth) and utilizes a structured alternative format, where children are presented with two statements (each describing a type of child) and must decide which kind of child he or she is most like. The child then decides whether the chosen description is ‘*really true for me* or *sort of true for me*.’ Internal consistency of the six subscales ranges from 0.73 to 0.91 (Muris et al., 2003; Harter, 2012). Specifically, internal consistency of the athletic competence subscale ranges from 0.76 to 0.91 (Harter, 2012). Internal consistency of the athletic competence subscale in the current sample was adequate (α = 0.83).

The *Child Outcome Rating Scales* (CORS; Duncan et al., 2003) is a four-item questionnaire used to assess feelings of wellbeing in children (aged 6–12) participating in an intervention. The scale requires rating across four domains—participants themselves and their family, school, and life in general. An overall “global distress” score can be calculated by summing subscales (Duncan et al., 2006). The scale has good internal consistency (α = 0.84) and moderate concurrent validity (*r* = 0.61) was established with the Youth Outcome Questionnaire (Duncan et al., 2006). Construct validity was established by Duncan et al. (2006) who found that all items of the CORS load on a common factor and the scale is also able to differentiate between normative and clinical populations.

#### Parent report measures

The Short Mood and Feelings Questionnaire for Parents (SMFQ-P; Angold et al., 1995) is the parent report version of the SMFQ, with the same 13-items to allow parent and child report comparison. The internal consistency was found to be good (α = 0.87) (Angold et al., 1995). Rhew et al. (2010) report the measure is able to adequately detect children at risk of depression. Internal consistency in the current sample was also good (α = 0.89).

The Spence Children's Anxiety Scale—Parent Version (SCAS-P; Spence, 1998) consists of 38-items to assess parent-reported child anxiety. Parents report on the frequency of symptoms via a four-point scale (*never, sometimes, often*, or *always*). Nauta et al. (2004) report the scale has demonstrated high internal consistency (α = 0.89). A moderate correlation (*r* = 0.66) was found between the parent and child versions of this scale. When compared to the Child Behavior Check List (CBCL; Achenbach and Rescorla, 2001), a screening tool for behavioral and emotional concerns, the SCAS-P has been found to have significantly higher correlation with the internalizing subscales (*r* = 0.55–0.59) than the externalizing subscales (*r* = 0.33–0.34) providing evidence for both convergent and divergent validity (Nauta et al., 2004). Internal consistency in the current sample is good (α = 0.90).

The parent version of the Strengths and Difficulties Questionnaire (SDQ-P; Goodman, 1999) provides an overall assessment of children's psychological adjustment. The SDQ-P is a 25-item scale comprising five subscales which assess emotional symptoms, conduct problems, hyperactivity/inattention, peer relationship problems, and pro-social behavior. Items are rated on a 3-point scale (*not true, somewhat true*, or *certainly true*). The first four subscales can be summed to generate a ‘total difficulties’ score, which has demonstrated good reliability (α = 0.82; Goodman, 2001). Internal consistency of subscales ranges from 0.59 to 0.81 (Hill and Hughes, 2007). The internal consistency of the total difficulties score in the current sample was good (α = 0.87).

### Procedure

Recruitment procedures included contact with primary schools, social media, and publicly placed fliers. Participants could also be referred to the program after contacting the Curtin University Psychology Clinic (Curtin Clinic) to request psychological services. Potential participants and their parent/s attended the Curtin Clinic for a screening interview conducted by Clinical Psychologist Trainees. Prior to the interview, participants were provided with an information sheet and consent forms. Following the interview, each child's capacity to consent to participate in the research, vulnerability, and suitability for the group were assessed. Eligible participants were then randomly allocated to the intervention or waitlist-control group via flip-of-coin. Children (and parents) in both groups were invited to complete pre-test measures online, via Qualtrics software in the Curtin University psychology computer lab prior to the intervention group's first session. Those participants who were unable to attend at this time were provided the link to the measures online and hardcopy questionnaires to return via post. Participants in the intervention group completed the CORS after each of the nine sessions. Post-test measures were then completed 9 weeks later following the last intervention group session. All participants completed these measures online and via hard copy in their own homes. Three months later, follow-up data was collected in the same way. Following completion of the measures at all time points, the parents of children with elevated scores on the SMFQ (>11) (Angold et al., 1995) and the SCAS (>42) (Barrett et al., 2005) were contacted in writing, under supervision from an experienced clinical psychologist, to advise of the results and provided with recommendations.

### Intervention

The 10 modules of the FFY3 program were delivered over 9 weeks, weekly sessions were completed over an hour, with module 8 and 9 completed together in an extended session to ensure the sessions concluded prior to school holidays. The program was delivered by two Clinical Psychologist Trainees, both of whom are accredited to facilitate Feelings and Friends (Year 3), and supervised by a clinical psychologist. Participants allocated to the waitlist-control group completed the program over 10 weeks, from March 2015. For the purpose of this study each FFY3 module was revised to include an activity from the Animal Fun program (see Table 1). The inclusion of activities and revision of module content were completed in consultation with Dr. Rosanna Rooney, a developer of the program, and Prof. Jan Piek, developer of Animal Fun.

**Table 1:** **Summary of the FFY3 Program Modules with the Included Animal Fun Activities**.

	**Topic**	**Content Summary**
1	Feelings and body clues	Increasing children's ability to identify and understand different feelings and learn about facial expressions. Animal Fun activity: Sing “If you're Happy and You know it” (social emotional development)
2	Feeling excited and proud	Identification of the feelings excited and proud; learning to recognize that body clues can help identify feelings. Animal Fun activity: Feeling Statues (social emotional development)
3	Feeling happy	Identify feeling happy; identify feelings in different situations and understand that feelings can vary in intensity. Animal Fun activity: Rocking Starfish (body management)
4	First aid for feelings	Introduction of the “First Aid Kit” as a tool to assist with uncomfortable feelings. Animal Fun activity: Flamingo (body movement)
5	First aid for scared and worried	Identifying the “body clues” that accompany feeling scared and worried. Animal Fun activity: Cat Stalking Mice (body management)
6	First aid for angry and sad	Identifying body clues associated with feeling angry and sad; learning to manage these feelings. Animal Fun activity: Friendly Fighting Antelopes (body management)
7	Other people's feelings	Learning to verbalize feelings and identify other people's feelings. Animal Fun activity: Animal Feely Cards (body management)
8	Caring about people's feelings	Learning how to be respectful and considerate of the feelings of other people. Animal Fun activity: Miss Mary Mack (fine motor planning)
9	How to be friendly	To identify friendly and unfriendly habits; learning to appreciate peers and show pro-social behavior. Animal Fun Activity: Push-Me Pull-You Race (body management)
10	Mad, sad and glad solutions	Identification of mad, sad and glad solutions; integration of all the skills learnt throughout the program. Animal Fun activity: Ants Working Hard (object control/cooperation)

### Data analysis

An independent sample *t*-test was applied to determine the significant baseline difference at pre-test between the intervention and control group for depression, anxiety, physical abilities and psychological difficulties. The psychometric data (SCAS, SMFQ, SPPC, SCAS-P, SMFQ-P, SDQ-P, SCAS-P Anxiety, SDQ-P externalizing, SDQ-P conduct) were analyzed with Generalized Linear Mixed Model (GLMM: Bryk and Raudenbush, 1987) as implemented through SPSS's (Version 22) GENLINMIXED procedure. GLMMs included one nominal random effect (participant) and three categorical fixed effects: Group (intervention vs. control), Time (pre-test, post-test, follow-up), and the Group × Time interaction. GLMM's “robust statistics” generally addresses violations of normality and homogeneity of variance. GLMM is less sensitive to participant attrition as it utilizes all data present at each assessment point, thus reducing sampling bias and the impact of missing data. To optimize the likelihood of convergence, a separate GLMM analysis was conducted for each outcome measure. Each GLMM assumed a normal probability distribution for the outcome and linked it to the fixed effects (group, time, Group × Time) with an identity function. If the outcome did not have a normal distribution, then the parameter estimates of the covariance matrix were computed with robust statistics. Unlike repeated measures ANOVA (or ANCOVA), GLMM does not rely on participants providing data at every assessment point; GLMM uses all the data present at each assessment point thereby reducing the impact of subject attrition on statistical power. Moreover, GLMM is robust to unequal group sizes, can deal with unequally spaced data collection points, does not require equal variances at each time point or equal covariances between all pairs of time points, and is able to account for correlations among repeated measurements.

Research using self-report measures of internalizing disorders has shown that among young children, anxiety and depression are distinct constructs (Murphy et al., 2000). As such, adjustment of the alpha level was not considered necessary. Effect sizes are reported following Cohen's conventions, where (*d*), small = 0.20; moderate = 0.50; large = 0.80; and partial eta squared ($\eta_{p}^{2}$), small = 0.01; moderate = 0.09; large = 0.25 (Cohen et al., 2003).

## Results

### Descriptive statistics

The current sample consisted of 24 children, 15 (62.5%) girls and 9 (37.5%) boys, with a mean age of 8.9 years (*SD* = 0.36). One parent of each participant (*n* = 24) also provided information. Attrition rates and further descriptive information is reported in Table 2.

**Table 2:** **Participant demographics**.

**Variable**		**Intervention (*n* = 12)**		**Waitlist control (*n* = 12)**	
		***M***	***SD***	***M***	***SD***
Child age (years)		8.8	0.43	8.6	0.23
		***N***	**%**	***N***	**%**
Gender Child	Male	6	50	3	25
	Female	6	50	9	75
Parent relationship to child	Mother	11	90	12	100
	Father	1	10	0	0
Attrition	Post-test	0	0	2	17%
	Follow-up	0	0	2	17%
	Total	0	0	4	33%

### Inferential statistics

An independent samples *t*-test yielded a significant pre-test difference between the intervention and control group for child-reported depression [*t*_(22)_ = 2.359, *p* = 0.028], with the intervention group reporting significantly higher depressive symptoms (*M* = 9.50, *SD* = 5.84) than the control group (*M* = 4.83, *SD* = 3.59). There were no other significant baseline differences between the two groups. Tables 3, 4 show means and standard deviations for outcome measures, as well as the main intervention effects.

**Table 3:** **Treatment outcome as a function of group**.

**Outcome measure and group**	**Pre-test**			**Post-test**			**Follow-up**					
	***M***	***SD***	***N***	***M***	***SD***	***N***	***M***	***SD***	***N***	**Group *F***	**Time *F***	**Group × Time *F***
SMFQ												
Intervention	9.50	5.84	12	8.42	6.37	12	7.55	7.09	11	5.80	0.88	0.35
Control	4.83	3.59	12	5.30	2.83	10	4.29	3.15	7	(*p* = 0.019)^*^	(*p* = 0.422)	(*p* = 0.706)
										(*d* = 0.15)	(*$\eta_{p}^{2}$* = 0.03)	(*$\eta_{p}^{2}$* = 0.01)
SCAS												
Intervention	41.30	18.27	12	32.67	9.90	12	26.73	13.43	11	2.40	2.57	0.52
Control	31.42	14.32	12	25.60	12.20	10	25.29	9.50	7	(*p* = 0.127)	(*p* = 0.035)	(*p* = 0.597)
										(*d* = 0.10)	(*$\eta_{p}^{2}$* = 0.08)	(*$\eta_{p}^{2}$* = < 0.01)
SPPC-ATH												
Intervention	17.32	3.47	10	17.82	3.79	11	17.64	3.78	12	1.50	1.86	0.49
Control	18.27	5.10	11	18.59	4.65	10	19.75	3.65	8	(*p* = 0.226)	(*p* = 0.164)	(*p* = 0.618)
										(*d* = 0.08)	(*$\eta_{p}^{2}$* = 0.06)	(*$\eta_{p}^{2}$* = 0.02)
SMFQ-P												
Intervention	8.17	4.80	12	4.42	4.03	12	5.17	3.71	12	0.03	8.20	3.34
Control	7.08	6.22	12	7.10	6.12	10	5.00	4.99	8	(*p* = 0.863)	(*p* = 0.001)^*^	(*p* = 0.042)^*^
										(*d* = 0.01)	(*$\eta_{p}^{2}$* = 0.21)	(*$\eta_{p}^{2}$* = 0.10)
SCAS-P												
Intervention	26.33	16.23	12	21.58	11.21	12	19.17	11.99	12	0.77	1.61	1.41
Control	25.67	10.11	12	28.00	15.33	10	26.00	12.98	8	(*p* = 0.383)	(*p* = 0.209)	(*p* = 0.251)
										(*d* = 0.06)	(*$\eta_{p}^{2}$* = 0.05)	(*$\eta_{p}^{2}$* = 0.04)
SDQ-P												
Intervention	15.25	4.61	12	12.08	3.78	12	12.83	4.24	12	0.09	4.43	0.77
Control	15.50	9.43	12	15.00	7.86	10	13.63	8.07	8	(*p* = 0.770)	(*p* = 0.016)^*^	(*p* = 0.469)
										(*d* = 0.02)	(*$\eta_{p}^{2}$* = 0.13)	(*$\eta_{p}^{2}$* = 0.03)

*SCAS, Spence Child Anxiety Scale; SMFQ, Short Mood and Feelings Questionnaire; SPPC-ATH, Self-Perception Profile for Children, Athletic Competence Subscale; SCAS-P, Spence Child Anxiety Scale–Parent Version; SMFQ-P, Short Mood and Feelings Questionnaire for Parents; SDQ-P, Strengths and Difficulties Questionnaire–Parent Version*.

* *p < 0.05*.

**Table 4:** **Treatment outcome for subscales with significant findings as a function of group**.

**Outcome measure and group**	**Pre-test**			**Post-test**			**Follow-up**					
	***M***	***SD***	***N***	***M***	***SD***	***N***	***M***	***SD***	***N***	**Group *F***	**Time *F***	**Group × Time *F***
SCAS-P Sep Anx												
Intervention	7.00	3.54	12	5.92	2.47	12	5.18	3.06	11	1.57	1.10	3.42
Control	6.58	3.75	12	5.50	3.28	10	4.42	2.99	7	(*p* = 0.215)	(*p* = 0.339)	(*p* = 0.039)^*^
										(*d* = 0.08)	(*$\eta_{p}^{2}$* = 0.04)	(*$\eta_{p}^{2}$* = 0.10)
SDQ-P Externalizing												
Intervention	7.75	3.11	12	5.00	1.86	12	6.25	2.42	12	0.783	1.88	7.09
Control	7.42	4.76	12	8.00	3.86	10	7.00	4.18	8	(*p* = 0.380)	(*p* = 0.162)	(*p* = 0.002)^*^
										(*d* = 0.06)	(*$\eta_{p}^{2}$* = 0.06)	(*$\eta_{p}^{2}$* = 0.19)
SDQ-P Conduct												
Intervention	2.33	1.72	12	1.25	1.14	12	1.92	1.00	12	1.731	2.64	5.70
Control	2.75	2.14	12	3.10	1.97	10	2.13	1.26	8	(*p* = 0.193)	(*p* = 0.080)	(*p* = 0.005)^*^
										(*d* = 0.08)	(*$\eta_{p}^{2}$* = 0.08)	(*$\eta_{p}^{2}$* = 0.16)

*SCAS-P Sep Anx, Separation Anxiety Subscale; SDQ-P Externalizing, Externalizing Subscale; SDQ-P Conduct, Conduct Subscale*.

* *p < 0.05*.

### Hypothesis testing

Results of GLMMs conducted are summarized in Table 3. The Group × Time interaction for child depression, as measured by the SMFQ, was not significant [*F*_(2, 58)_ = 0.35, *p* = 0.706, $\eta_{p}^{2}$ = 0.01]. The main effect of group, however, was significant [*F*_(1, 58)_ = 5.80, *p* = 0.019, *d* = 0.15], indicating that the intervention group had significantly higher levels of depressive symptoms than the control group. There was no Group × Time interaction for child anxiety as measured by the SCAS [*F*_(1, 58)_ = 2.40, *p* = 0.127, $\eta_{p}^{2}$ = < 0.00] or for self-perceived physical ability as measured by the SPPC Athletic Competence subscale [*F*_(2, 56)_ = 0.49, *p* = 0.618, $\eta_{p}^{2}$ = 0.02].

**Figure 2:**
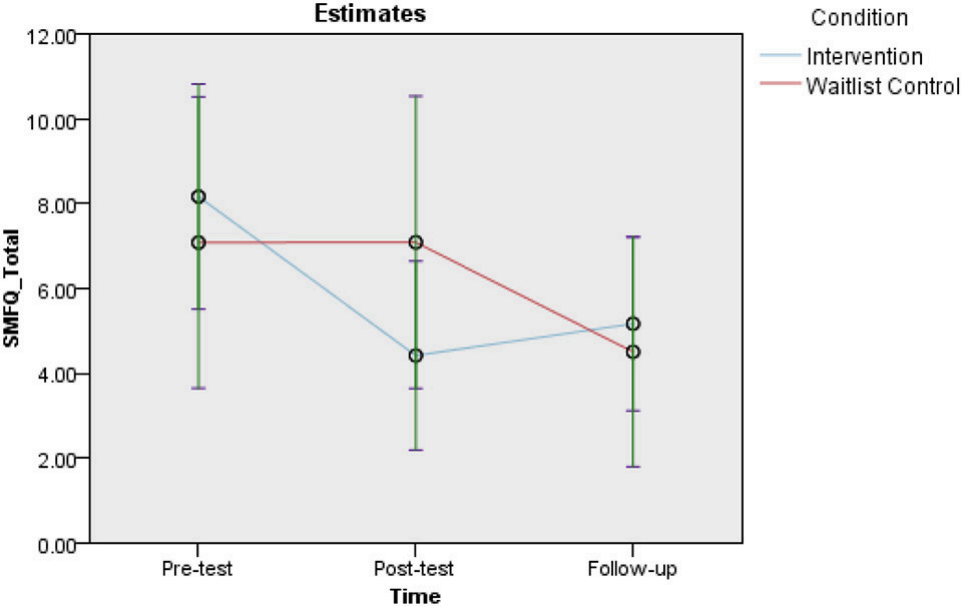
**Participant's scores on the SMFQ-P at each assessment point**.

There was a significant Group × Time interaction for parent-reported depression, as measured by the SMFQ-P [*F*_(2, 60)_ = 3.34, *p* = 0.042, $\eta_{p}^{2}$ = 0.10]. Least significant difference (LSD) *post-hoc* contrasts indicated a significant decrease in depressive symptoms from pre-test to post-test [*t*_(60)_ = 3.32, *p* = 0.002, *d* = 0.86] and from pre-test to follow-up [*t*_(60)_ = 2.86, *p* = 0.006, *d* = 0.74] for the intervention group. The control group showed a significant change from pre-test to follow-up [*t*_(60)_ = 2.58, *p* = 0.012, *d* = 0.67]. These findings are displayed in Figure 2.

There was no significant Group × Time interaction for parent-reported child anxiety, as measured by the SCAS-P [*F*_(2,60)_ = 1.41, *p* = 0.251, $\eta_{p}^{2}$ = 0.04]. There was, however, a significant Group × Time interaction for parent-reported separation anxiety as measured by the separation anxiety subscale of the SCAS-P [*F*_(2,60)_ = 3.42, *p* = 0.039, $\eta_{p}^{2}$ = 0.10]. LSD *post-hoc* contrasts indicated a significant decrease pre-test to follow-up [*t*_(60)_ = 2.22, *p* = 0.030, *d* = 0.57] and post-test to follow-up decrease [*t*_(60)_ = 2.26, *p* = 0.028, *d* = 0.58] for the intervention group only.

There was no intervention effect for parent-reported child adjustment, as measured by the SDQ [*F*_(2,58)_ = 0.77, *p* = 0.469, $\eta_{p}^{2}$ = 0.03]. However, there was a significant main effect of time [*F*_(2, 58)_ = 4.43, *p* = 0.016, $\eta_{p}^{2}$ = 0.13], indicating that both groups changed significantly over time, and at the same rate. *Post-hoc* LSD contrasts of the SDQ indicated a significant decrease in difficulties from pre-test to post-test [*t*_(58)_ = 2.86, *p* = 0.006, *d* = 0.74] and from pre-test to follow-up [*t*_(58)_ = 2.35, *p* = 0.022, *d* = 0.61]. There was also a significant Group × Time interaction on the externalizing subscale of the SDQ [*F*_(2,60)_ = 7.091, *p* = 0.002, $\eta_{p}^{2}$ = 0.19]. LSD *post-hoc* contrasts indicated a significant pre-post decrease for the intervention group [*t*_(60)_ = 4.75, *p* = 0.000, *d* = 1.21], that was maintained at follow-up [*t*_(60)_ = 2.05, *p* = 0.045, *d* = 0.52] while the control group showed no significant changes across time. Also observed was a significant Group × Time interaction on the conduct problems subscale of the SDQ [*F*_(2, 60)_ = 5.70. *p* = 0.005, $\eta_{p}^{2}$ = 0.16]. LSD *post-hoc* contrasts indicated a significant pre-post decrease for the intervention group [*t*_(60)_ = 2.60, *p* = 0.012, *d* = 0.66] and a significant post to follow-up decrease for the control group [*t*_(60)_ = 2.11, *p* = 0.039, *d* = 0.54].

Evaluation of the CORS showed a significant main effect for time [*F*_(8, 92)_ = 3.18, *p* = 0.003, $\eta_{p}^{2}$ = 0.22], indicating a significant difference *between at least two time points*. LSD contrasts showed a significant increase from session one to session two [*t*_(92)_ = 2.31, *p* = 0.023, *d* = 0.48]; all other contrasts were non-significant. Table 5 shows means and standard deviations for outcome measures.

**Table 5:** **Treatment outcome for the CORS (Intervention group)**.

**Outcome measure**	***M***	***SD***	***N***
Time 1	27.63	12.73	13
Time 2^*^	29.73	9.58	13
Time 3	27.78	13.50	13
Time 4	29.52	14.66	13
Time 5	29.59	12.09	13
Time 6	26.48	14.33	13
Time 7	28.85	13.59	13
Time 8	27.60	15.05	13
Time 9	30.93	11.54	13

* p < 0.05

## Discussion

The aim of the current study was to evaluate the efficacy of the revised Feelings and Friends (Year 3) program (FFY3) via a randomized controlled trial with 8 and 9 year-old children and their parents. Examination of the hypotheses revealed support for the efficacy of the program in reducing parent-reported child depression (H3a) with a moderate effect size. Contrary to predictions, results indicated no significant short-term or 3-month follow-up intervention effects for child-reported depression (H1a), anxiety (H1b), and self-perceived athletic competence (H2) or parent-reported anxiety (H3b) and overall psychological adjustment (H3c). Examination of subscales provided support for the efficacy of the program in reducing parent-reported separation anxiety, externalizing symptoms, and conduct problems. A further outcome suggested that the program has the potential to improve children's well-being (H4). Overall, the findings from this study indicate that FFY3 is a promising prevention for internalizing and externalizing symptoms in 8 and 9 year-old children.

In contrast to predictions, there were no intervention effects for child-reported depression (H1a) or anxiety (H1b). These results are similar to prior clinical trials, which have failed to find significant reductions in child-reported symptomology using clinical measures (e.g., Pophillat, 2007; Green, 2008; Fresel, 2013). In fact, numerous trials of prevention programs for children have failed to find *post-test* reductions in child-reported depressive and anxious symptoms (e.g., Dadds et al., 1997; Lock and Barrett, 2003; Hudson et al., 2009). An exception to this is the reductions in depressive symptoms (*d* = 0.54) reported by Kam et al. (2004) in a study of the PATHS program. Interestingly, some studies have reported no reductions in depressive and anxious symptoms at post-test have found them at long-term (12-month) follow-up (Dadds et al., 1997; Lock and Barrett, 2003). While the current study included a longer-term (3-month) follow-up than prior Feelings and Friends trials, future research could include 6–12-month follow-up to increase the likelihood of detecting significant effects. Other researchers have noted that young children's ability to report on their own emotional states is limited (e.g., Dadds et al., 1997), which may explain the lack of intervention effects for child-reported outcomes in this study.

By including Animal Fun activities, it was predicted that participants' self-perceived physical competence would improve, having flow on benefits to reducing the symptoms of internalizing disorders. However, results showed no intervention effects for self-perceived physical ability (H2). In its original form, Animal Fun was to be delivered 4 days per week, for at least 10 weeks (Piek et al., 2010). Thus, the inclusion of only one activity per session may not have been adequate dosage to improve skills or perception of competence in this area. Animal Fun activities were also originally developed for children aged 4–6 years (Piek et al., 2012a). Participants in the intervention group had a mean age of 8 years, 9 months so it is possible that the activities may not have been developmentally appropriate. Future research could investigate the appropriateness of Animal Fun activities to this older age bracket.

A key finding of the current research was the observed intervention effect for parent-reported child depression, supporting Hypothesis 3a, and consistent with a previous trial of the program (Fresel, 2013). Taken together these findings provide promising support for the efficacy of the Feelings and Friends program at reducing parent-observed child depressive symptoms. These findings align with those of previous trials of universal prevention programs which have shown reductions in child depression (e.g., Kam et al., 2004), however, considering the labor- intensive implementation required for some of these programs (e.g., 1 year) Feelings and Friends could be considered superior with regard to time efficiency. Interestingly the control group's follow-up scores showed marked improvement from pre-test, which was not evident at post-test. The difference here may be explained by the time of year data collection occurred. At follow-up, children had started a new year at school, which may have been a positive event in their lives. A further explanation on the control group's improvement is that children who scored above the anxiety and depression cut-off point at pre-test were confidentially referred for assistance to alleviate the symptoms.

In line with Fresel's (2013) findings there was no significant reduction in parent-reported anxiety, Hypothesis 3b was not supported. This differs from findings of prior trials of this program (Pophillat, 2007; Green, 2008) and other prevention programs (e.g., Hudson et al., 2009) that have found significant reductions in parent-reported anxiety. An interesting finding from the current research was the significant intervention effect for parent-report symptoms of separation anxiety disorder (SAD). It is possible that due to the characteristic symptoms of excessive distress and refusal (American Psychiatric Association, 2013), it may have been easier for parents to identify a reduction in SAD symptoms within the given time period, compared to reductions in less visceral symptoms that characterize internalizing problems (e.g., withdrawal).

The predicted reduction in psychological difficulties (H3c) was not supported, in opposition to the findings of Green (2008). However, in contrast to Green's open trial design the current study utilized a randomized controlled trial design, increasing internal validity and giving credibility to the findings. Examination of the SDQ subscales revealed a significant reduction in externalizing symptoms and conduct problems for children in the intervention group. These findings align with research on an Aussie Optimism program for 9–10 year olds, which found a reduction in externalizing symptoms maintained at 30-month follow-up (Rooney et al., 2013). Trials of the PATHS program, which targets externalizing symptoms, have shown reductions in child-reported aggression (Crean and Johnson, 2013) and in teacher-reported externalizing symptoms (Kam et al., 2004). Considering the labor intensive implementation required for PATHS, the findings of the current research are promising for participation in the comparatively less labor intensive FFY3, for a significant reduction in externalizing symptoms and conduct problems. Future research may wish to investigate if there is a link between these reductions and the inclusion of Animal Fun activities.

Finally, this study aimed to replicate the findings of Green's (2008) open trial, in relation to improvements in global distress, measured via the CORS. Green compared pre-post completion of the CORS for children participating in the program, finding a significant improvement. As the CORS is designed to track the progress of children participating in an intervention, session-to-session, comparison of results in this way was considered more meaningful than from pre-post. Hypothesis 4 was partially supported in the finding that participants rating of global distress improved from session one to session two. Session one focused on basic skills to identify a range of feelings compared with session two which focused on body clues associated with feeling excited and proud, and included an activity to plan a group party. The content of session two may have served to excite the children and improve their mood which resulted in significant improvement in the CORS.

The small effect sizes observed in the current findings are corroborated by prior research on universal child prevention programs which has shown that the average effect sizes for internalizing symptoms from pre- to post-treatment and follow-up are small, while still yielding significant reductions in symptomology (Stice et al., 2009). As this program was developed for a normative sample, recruitment of participants was not based on clinical cut-offs and a high-risk sample was not sought out. If a high-risk sample had been selected, larger effect sizes may have been observed (Horowitz and Garber, 2006). Nevertheless this study produced reductions in internalizing symptoms, with small effect sizes, which holds clinical significance.

### Limitations of the current study and future research

Some methodological limitations should be considered when interpreting the current results. Low statistical power, as a result of the small sample size, is likely to have contributed to the non-significant findings. To address this limitation, recruitment of bigger sample size would be highly recommended, however, Cuijpers (2003) found that universal prevention programs required tens of thousands of participations to be feasible and sufficient. Selective and indicative prevention programs are the alternative options available, however these lack a major advantage of universal prevention programs, in delivery to a broad population (e.g. via delivery in a school) regardless of risk or diagnosis. Future research may wish to facilitate multiple intervention groups on different days of the week, as the day and time of the program may have inhibited recruitment. Given the revision of the program involved the removal of various activities, it is possible that FFY3 failed to deliver a dosage large enough to further reduce internalizing symptoms. In the future, session times could be increased to accommodate additional activities. Other potential drawbacks were noted in data collection. The majority of pre-test data was collected in group sessions, creating the potential for participants to distract each other. Despite the use of age appropriate self-report measures, an unexpected issue was the impact of children's reading ability. Several children required assistance to complete self-report measures, which may have influenced their responses. Future research may wish to assess the reading ability of children and utilize more robust age-specific assessment tools that suits the children's capabilities. Anderson and Pearson (1984) asserted that reading ability and comprehension is a constructive process that involves explicit use of prior knowledge to assist in understanding and interpreting new information presented in the text. Use of a cognitive assessment enriched with graphics and pictures is recommended as children tend to relate better to images than linguistic text. According to Freed et al. (2015) pictures facilitate and enhance the mental representation process, thus resulting in less burden on working memory. Cain et al. (2004) stated that the working memory process is highly influenced by the resources presented (e.g., visual or spoken) and it can affect children's reading comprehension level. Visual representation will most likely increase the likelihood that students provide more accurate responses on the questions asked. A long and wordy self-report assessment may hinder young children's task concentration and focus as they have short attention spans and limited vocabulary, syntax, and knowledge (Fang, 1996).

As the FFY3 program was developed for universal prevention, children were not excluded from participating on the basis of clinical cut-offs or disability diagnosis. In spite of random allocation, participants in the intervention group included two children with a diagnosis of Autism Spectrum Disorder and two children with learning disorders, and the group was also shown to have significantly higher levels of depressive symptoms than the control group at baseline. While the statistical procedures utilized accounted for differences between the groups at baseline, the issues impacting on children in the intervention group may have impeded participants' abilities to engage with the content of the modules and/or affected implementation and thus influenced the findings. Future research may wish to investigate the efficacy of FFY3 for children with developmental disabilities.

Implementation of FFY3 in a clinic setting rather than a school may have limited the effects observed. The program was designed for implementation in a classroom as part of the primary school health education curriculum (Rooney et al., 2014). Compared to the contact teachers have with children, the program facilitators had drastically reduced opportunity (i.e., 1 hour per week) to reinforce the principles. Future research could explore the differences between teacher and psychologist facilitators and small vs. large groups. The costs and benefits of employing psychologists to deliver a universal program also need to be weighed up. In addition, the use of teacher-reported outcomes may also provide insight into changes in child behavior.

## Conclusion

The current study has contributed to the body of research investigating the efficacy of programs to prevent the development of internalizing disorders in young children. Results indicated reductions in parent-reported child depression in a sample of 8 and 9 year-old children from schools in Perth, Western Australia. Furthermore, this study provides evidence for a reduction in parent-reported separation anxiety, externalizing symptoms, and conduct problems. These results are important as they provide evidence that FFY3, a minimally labor intensive program, is sufficient to produce reductions in the symptoms of internalizing and externalizing disorders. Further research is needed to investigate the contribution of motor coordination activities and their impact on the program. Given its brevity, FFY3 has the potential to be disseminated widely in school settings, providing a cost-effective intervention to reduce emotional and behavioral problems in children. Due to its universal implementation, this program may prove to reduce stigma as children are not singled out and also ensure that “at risk” children are not overlooked. Preventing the development of emotional problems in young children is likely to manifest positive effects for the individual and wider community.

## Ethics statement

Curtin University Ethics committee. Written consents for the young children were obtained from the parents before the intervention study. Potential participants and their parents attended the Curtin Clinic for a screening interview conducted by clinical psychologist trainees. Prior to the interview participants were provided with an Information sheet and consent forms. Following the interview, each child capacity to consent to participate in the research, vulnerability, and suitability for the group were assessed.

## Author contributions

RT: Conducted the intervention study and wrote the manuscript. KM: Conducted the intervention study and assisted in writing the manuscript. RR: Co-author of the intervention program and contribution to introduction and discussion sections of the manuscript. SH: Co-author of the intervention program and contribution in the results and discussion sections of the manuscript. RK: Methodology and data analysis.

## Funding

Mental Health Commission Western Australia (Grant number MHC69) and School of Psychology and Speech Pathology, Curtin University.

### Conflict of interest statement

The authors declare that the research was conducted in the absence of any commercial or financial relationships that could be construed as a potential conflict of interest.
